# Group structure and kinship in beluga whale societies

**DOI:** 10.1038/s41598-020-67314-w

**Published:** 2020-07-10

**Authors:** Greg O’Corry-Crowe, Robert Suydam, Lori Quakenbush, Thomas G. Smith, Christian Lydersen, Kit M. Kovacs, Jack Orr, Lois Harwood, Dennis Litovka, Tatiana Ferrer

**Affiliations:** 1 Harbor Branch Oceanographic Institute, Florida Atlantic University, 5600 US. 1 North, Fort Pierce, FL 34946 USA; 2 North Slope Borough Department of Wildlife Management, 1274 Agvik Str., Utqiagvik, AK 99723 USA; 3 Alaska Department of Fish and Game, 1300 College Road, Fairbanks, AK 99701 USA; 4 Eco Marine Corporation, 5694 Camp Comfort Road, Beaulac-Garthby, QC G0Y 1B0 Canada; 5 Norwegian Polar Institute, Framsenteret, Hjalmar Johansens Gate 14, 9296 Tromsø, Norway; 6 Fisheries and Oceans Canada, 501 University Crescent, Winnipeg, MB R3T 2N6 Canada; 7 Fisheries and Oceans, Canada, 301-5204 50th Ave., Yellowknife, NT X1A 1E2 Canada; 8 Office of the Governor and Government of the Chukotka Autonomous Region, Str. Bering 20, Anadyr, 689000 Russia

**Keywords:** Ecology, Evolution

## Abstract

Evolutionary explanations for mammalian sociality typically center on inclusive-fitness benefits of associating and cooperating with close kin, or close maternal kin as in some whale societies, including killer and sperm whales. Their matrilineal structure has strongly influenced the thinking about social structure in less well-studied cetaceans, including beluga whales. In a cross-sectional study of group structure and kinship we found that belugas formed a limited number of distinct group types, consistently observed across populations and habitats. Certain behaviours were associated with group type, but group membership was often dynamic. MtDNA-microsatellite profiling combined with relatedness and network analysis revealed, contrary to predictions, that most social groupings were not predominantly organized around close maternal relatives. They comprised both kin and non-kin, many group members were paternal rather than maternal relatives, and unrelated adult males often traveled together. The evolutionary mechanisms that shape beluga societies are likely complex; fitness benefits may be achieved through reciprocity, mutualism and kin selection. At the largest scales these societies are communities comprising all ages and both sexes where multiple social learning pathways involving kin and non-kin can foster the emergence of cultures. We explore the implications of these findings for species management and the evolution of menopause.

## Introduction

Interpreting gregarious behaviour in terms of cooperative strategies that maximize individual fitness, Hamilton^1^ developed the theory of kin selection. This theory uses the concept of inclusive fitness to explain the evolution of social organization and cooperation where individuals indirectly enhance their fitness through positive effects on the reproduction of relatives. However, individuals can also derive benefits from associations with unrelated individuals where cooperation is conditional on the behaviour of the companion (i.e., reciprocity^2,3^), always yields the highest benefit (i.e., mutualism^4,5^) or results in shared fitness advantages from helping increase group size (i.e., group augmentation^6,7^) which, for example, could lead to more effective group defense. In fluid aggregations, such as herds or flocks, benefits to the individual alone (e.g., reducing personal predation risk at the expense of other group members) may drive the tendency to associate with conspecifics^8,9^. Thus, resolving the genetic relationships among group members is central to understanding the advantages of group living, the emergence of cooperative behaviour, and the evolution of social organization. The recent re-emergence of arguments for group selection theory, where kinship plays a minor role in social evolution^10,11^, and the debate these arguments have elicited^12^, as well as the growing evidence for culture in non-primate species^13–15^ (defined as the acquisition or inheritance of knowledge or behaviours from conspecifics through social learning^13^), has further heightened the interest in the role of kinship in the characteristics, dynamics and function of groups in social species.

Many aspects of beluga whale (*Delphinapterus leucas*) behavioural ecology, including their highly gregarious behaviour^16^ and sophisticated vocal repertoires^17,18^, associated with a diverse suite of interactive behaviours^19,20^, suggest that this arctic cetacean lives in complex societies. Belugas exhibit a wide range of grouping patterns from small groups of 2–10 individuals to large herds of 2,000 or more, from apparently single sex and age-class pods to mixed-age and sex groupings, and from brief associations to multi-year affiliations^16,21–23^. This variation suggests a fission–fusion society where group composition and size are context-specific, but it may also reflect a more rigid multi-level society comprised of stable social units that regularly coalesce and separate. The role kinship plays in these groupings is largely unknown.

It has been postulated that beluga whale group structure centres around females with their calves of different ages^16,21^ and is similar to the group structure in killer whales (*Orcinus orca*) and some other odontocete whale species^21,24^ that primarily comprise closely related individuals from the same maternal lineage^25–29^. Group structure is quite different in other odontocetes, such as the bottlenose dolphin (*Tursiops* spp.), where grouping patterns vary from all-male alliances and female bands to mixed groups of varying size and stability^30,31^. While matrilineal affiliations exist, bottlenose dolphin groups are not strictly matrilineal and the extent of kinship within groups varies dramatically^32–34^.

Genetic studies have revealed significant geographic partitioning of mtDNA lineages in beluga whales^35–38^ and found that relatives sometimes travel together within large migrating herds or occur in close temporal proximity throughout the migratory cycle more frequently than expected by chance^39,40^. These findings, in concert with the discovery of closely related individuals returning to the same summering location years and even decades apart, are compelling evidence of natal philopatry to migration destinations where the strong mother–calf bond may facilitate the cultural learning of migration routes^40^. However, it must be noted that herds also contain large numbers of unrelated individuals^39,40^ and the potential preferential association of matrilines (or even just close kin) beyond mother–calf pairs within these large seasonal aggregations has not been investigated. Furthermore, there is almost no information on the possible role of kinship in smaller groupings. And yet, the model of a stable matrilineal group as the cornerstone of beluga society is often used as a social framework to interpret other aspects of beluga whale behaviour and ecology^24,41,42^.

**Figure 1:**
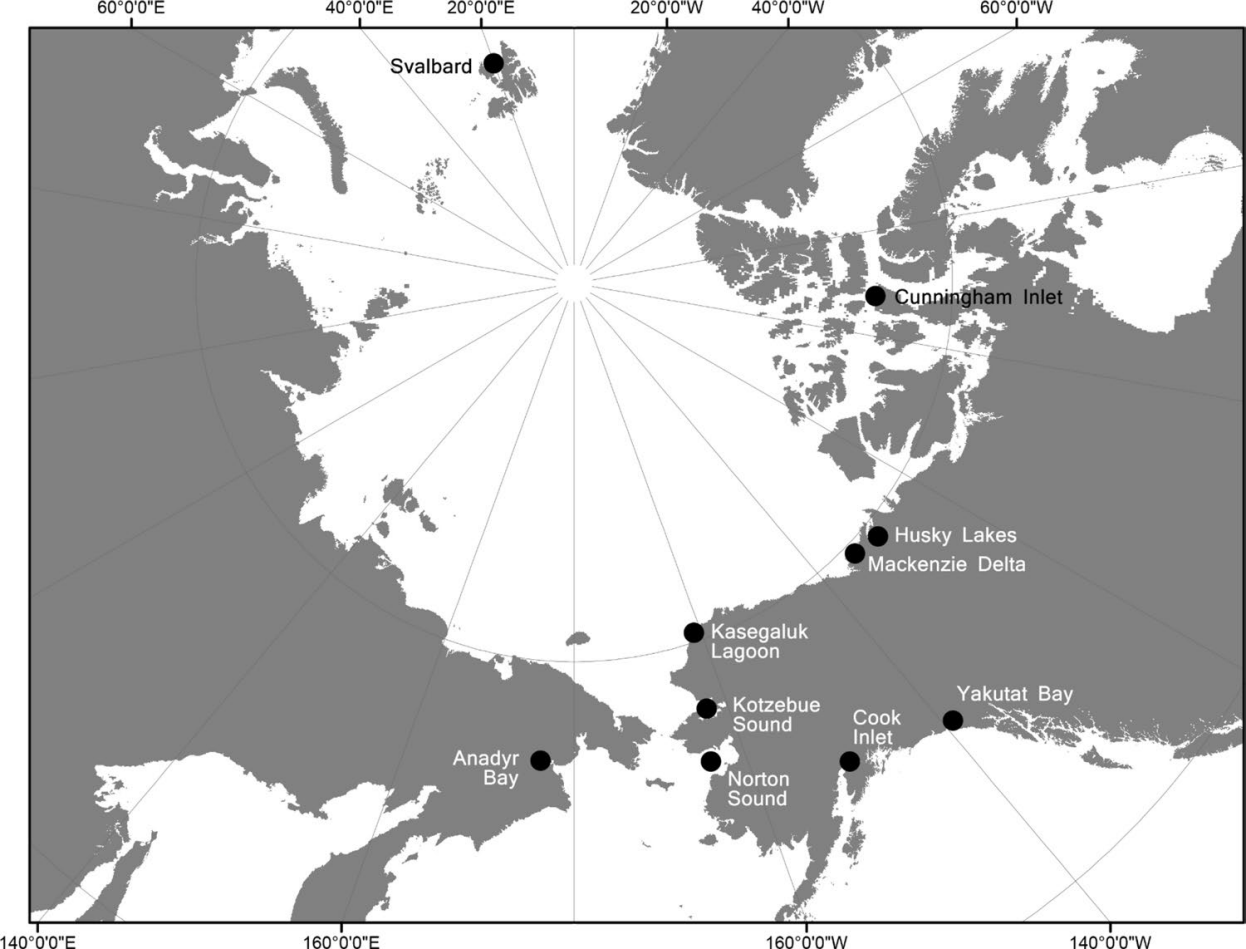
Map of the Arctic showing the ten locations across the beluga whales’ range where group structure, behavior, dynamics and kinship were investigated. In some locations only limited field data was collected. The map was generated based on a publicly available ArcMap polar projection document using ArcGIS 10.1 (www.esri.com).

In this study we used field observations, mtDNA profiling, and multi-locus genotyping of beluga whales to address fundamental questions about beluga group structure, and patterns of kinship and behaviour that provide new insights into the evolution and ecology of social structure in this Arctic whale. The study was conducted at ten locations, in different habitats, across the species’ range, spanning from small, resident groups (Yakutat Bay) and populations (Cook Inlet) in subarctic Alaska to larger, migratory populations in the Alaskan (Kasegaluk Lagoon, Kotzebue Sound, Norton Sound), Canadian (Cunningham Inlet, Mackenzie Delta, Husky Lakes) and Russian (Gulf of Anadyr) Arctic to a small, insular population in the Norwegian High Arctic (Svalbard) (Fig. 1).

We investigated whether there are basic types of groupings and association patterns in beluga whales that are consistently observable within and across populations and habitats, and if so, to what degree are they kin-based. The following seven hypotheses were tested: (H_1_) beluga whales form a limited number of distinct group types observable across varied locations and habitats; (H_2_) certain behaviours are more prevalent in particular group types; (H_3_) beluga whale groups are predominantly kin-based, comprising high proportions of close relatives, (H_4_) beluga whale groups are matrilineal, comprised of close maternal relatives, (H_5_) females are more related than males within groupings; (H_6_) larger herds are composed of multiple distinct matrilineal groupings; and (H_7_) adult-only groups are exclusively comprised of males. We compare our findings with current knowledge on social structure of other odontocete whales. We interpret our results in the context of existing theories on the evolution of social organization and discuss their implications for beluga whale management in a changing Arctic including how social disruption might influence culture and population recovery.

## Results

### Grouping patterns and behaviour

Seven distinct group types were identified in beluga whales, two of which fell under the definition (see “Methods”) of a *herd* (i.e., > 50 animals) and five that were defined as *social groups* (i.e., ≤ 50 animals; Table 1). These group types were observed repeatedly within and across multiple locations and habitats (Table 1). The five types of social group were: (A) adult–calf dyads, (B) groups consisting only of adults with calves, (C) groups of juveniles only, (D) groups of adults only, and (E) mixed-age groups. The two types of herd were (F) adult-only herds and (G) mixed-age herds. We further distinguished two other grouping types from the mixed-age herd type: (H) daily aggregations, and (I) multi-day aggregations. These latter groupings were essentially mixed-age herds that were not under continuous observation over the period of tissue sampling (i.e., a single day and several days, respectively) and therefore simultaneous association among all sampled whales in the grouping could not be affirmed. A possible sixth social group type was observed—an adult with two calves of differing ages—tentatively termed a triad, though this possible group type was observed only once (group type A_1_, Table 1). At one location, Kasegaluk Lagoon (Fig. 1), whales that were in a herd were slowly driven in a set direction by a number of small boats for several hours prior to observation and sampling which may have influenced herd composition. However, we assume that these mixed-age herds were predominantly natural associations.

**Table 1:** Beluga whale association patterns and behavior at ten locations that span different populations and habitats across the species range.

	Group type	Location	Date	Description	Behaviour*			Field analysis		Genetic analysis		
					Category	Details	Likely function	Group size	Group ages**	Sample size	Sex	Sample ages**
A	Adult–calf dyad	Svalbard	8/23/1998	Summer aggregation	T, O	Directed movement, close association	Travel, natal care	2	A, C	2	F, M	A, C
	Adult–calf dyad	Svalbard	8/23/1998	Summer aggregation	T, O	Directed movement, close association	Travel, natal care	2	A, C	2	F	A, C
	Adult–calf dyad	Svalbard	8/24/1998	Summer aggregation	T, O	Directed movement, close association	Travel, natal care	2	A, C	2	F	A, C
	Adult–calf dyad	Svalbard	8/21/1999	Summer aggregation	T, O	Directed movement, close association	Travel, natal care	2	A, C	2	F	A, C
	Adult–calf dyad	Norton Sound	9/23/1995	Fall movements	T, O	Directed movement, close association	Travel, natal care	2	A, C	2	F, M	A, C
	Adult–calf dyad	Cook Inlet	10/31/2001	Resident population	T, O	Directed movement, close association	Travel, natal care	2	A, C	2	F	A, C
	Adult–calf dyad	Mackenzie delta	8/1/1997	Summer aggregation	T, O	Directed movement, close association	Travel, natal care	2	A, C	2	F, M	A, C
	Adult–calf dyad	Cunningham Inlet	7/25/1998	Summer aggregation	T, O	Directed movement, close association	Travel, natal care	2	A, C			
A_1_	Triad	Cunningham Inlet	7/25/1998	Summer aggregation	T	Large white, neonate, 2/3 grey: in association	Travel, natal care	3	A, C			
B	Adult–calf group	Cunningham Inlet	7/26/1998	Summer aggregation	S	Contact, turning, loose group	Social interaction, creshe	15–20	A, C	3	F	A
	Adult–calf group	Cunningham Inlet	7/23/1998	Summer aggregation	T	Adult with 3/4 grey-white and 1/2 grey	Travel	3	A, J, C			
	Adult–calf group	Cunningham Inlet	7/23/1998	Summer aggregation	T	3 adults w. calves	Travel	6	A, C			
	Adult–calf group	Cunningham Inlet	7/23/1998	Summer aggregation	T, O	2 adults with neonates, close association	Travel, natal care	4	A, C			
	Adult–calf group	Cunningham Inlet	7/24/1998	Summer aggregation	S, O	9 adult w. calves, rubbing on substrate, swimming upside down, spy hopping	Social interaction, molting	18	A, C			
	Adult–calf group	Cunningham Inlet	7/25/1998	Summer aggregation	T	5 adults w. calves, loose group in close association, directed movement	Travel, natal care	10	A, C			
C	Juveniles only group	Cunningham Inlet	7/24/1998	Summer aggregation	S, O	Contact, rapid turning, bubble blasts, vocalizations, chase, rubbing	Social interaction, play, molt	3	J	3	F	J
	Juveniles only group	Cunningham Inlet	7/23/1998	Summer aggregation	S	Contact, twisting, spy hopping	Social interaction, play	2	J			
	Juveniles only group	Cunningham Inlet	7/23/1998	Summer aggregation	T, S	Swim in unison	Social interaction, travel, dominance	4	J			
	Juveniles only group	Cunningham Inlet	7/25/1998	Summer aggregation	S	Active interactions	Social interaction, play	3	J			
	Juveniles only group	Cunningham Inlet	7/26/1998	Summer aggregation	S	Individuals moving between 4 sub-groups, contact, upsidedown, aerial, rubbing,	Social interaction, play, molt	17	J			
D	Adults only group	Cunningham Inlet	7/25/1998	Summer aggregation	S, O	Rubbing, swim in close formation, aggression to others—observed for 3 h	Social interaction, dominance, molt	7	A	5	M	A
	Adults only group	Cunningham Inlet	7/27/1998	Summer aggregation	S	Swim in close formation, aggression to others	Social interaction, dominance	2	A	2	M	A
	Adults only group	Svalbard	8/4/1997	Summer aggregation and fall movements	T	Directed movement, travelled together for months	Travel	3	A	3	M	A
	Adults only group	Svalbard	8/12/1997	Summer aggregation	T	Directed movement	Travel	2	A	2	M	A
	Adults only group	Mackenzie Delta	29/7/1997	Summer aggregation	T	Directed movement, synchronized summer movements	Travel	3	A	2	M	A
	Adults only group	Mackenzie Delta	31/7/1997	Summer aggregation	T	Directed movement, synchronized summer movements	Travel	~ 15	A	2	M	A
	Adults only group	Cunningham Inlet	7/23/1998	Summer aggregation	T	Directed movement, head up, avoided by other whales	Travel, dominance	5	A			
	Adults only group	Cunningham Inlet	7/23/1998	Summer aggregation	T, S	Swim in unison-parallel, avoided by other whales	Social interaction, travel, dominance	10	A			
	Adults only group	Cunningham Inlet	7/24/1998	Summer aggregation	S, T	Patrol beach back and forth	Social interaction, travel, dominance	11	A			
	Adults only group	Cunningham Inlet	7/25/1998	Summer aggregation	T, O	Slowly swimming, rubbing on substrate	Travel, molt	12	A			
	Adults only group	Cunningham Inlet	7/26/1998	Summer aggregation	S	Contact, lateral ventrum presentation, chasing off smaller whales	Social interaction, dominance, sexual (?)	2	A			
	Adults only group	Cunningham Inlet	7/26/1998	Summer aggregation	T, S	Audible vocalizations, slow, coordinated movements	Social interaction, travel	2	A			
	Adults only group	Cunningham Inlet	7/26/1998	Summer aggregation	S	Rolling, etc. chasing off other whales	Social interaction	8	A			
	Adults only group	Cunningham Inlet	7/27/1998	Summer aggregation	T, S	Loose group; directed movement, rolling, spyhop, 'foghorn' vocal	Travel, social interaction	12	A			
	Adults only group	Cunningham Inlet	7/27/1998	Summer aggregation	S	Patrolling shore, contact, aggression to others, aerial flipper,	Socisal interaction, dominance, sexual (?)	3	A			
	Adults only group	Cunningham Inlet	7/28/1998	Summer aggregation	S	Chasing Smaller whales, vocalizations	Social interaction, dominance	3	A			
	Adults only group	Cunningham Inlet	7/27/1998	Summer aggregation	T	Directed movement in close association	Travel	2	A			
E	Mixed age group	Cunningham Inlet	7/24/1998	Summer aggregation	S	Contact, rapid turning, bubble blasts, vocalizations	Social interaction	5	A, J	2	F	A, J
	Mixed age group	Cunningham Inlet	7/28/1998	Summer aggregation	S	Contact, rapid turning	Social interaction	~ 50	A, J	6	F, M	A, J
	Mixed age group	Svalbard	8/22/1998	Summer aggregation	T	Directed movement	Travel	5	A, J	5	F, M	A, J
	Mixed age group	Anadyr Bay	7/18/2001	Summer aggregation	T, S	Directed movement, contact, rapid turning	Travel, social interaction	_	A, J, C	11	F, M	A, J, C
	Mixed age group	Yakutat Bay	5/19/2005	Resident group	T, M, S	Directed movement, milling, social interaction	Travel, social interaction, possible feed	6–8	A, J	2	M, –	A
	Mixed age group	Yakutat Bay	5/19/2008	Resident group	T	Directed movement	Travel	5–8	A, J	2		A, J
	Mixed age group	Cunningham Inlet	7/26/1998	Summer aggregation	T	2 adult whites w. adult–calf pair: in association	Travel	4	A, C			
	Mixed age group	Cunningham Inlet	7/27/1998	Summer aggregation	T, S	Directed movement, range of activities	Travel, social interaction, play	3				
	Mixed age group	Cunningham Inlet	7/29/1998	Summer aggregation	T, S	2 adult whites loosely associating w. and following adult-neonate pair	Travel, pursuit-courtship	4	A, C			
F	Adults only herd	Kasegaluk Lagoon	6/26/1998	Spring migration, staging	T, M, S	Directed movement, milling, close association, turning	Migration, social interaction	_	A	54	M	A
	Adults only herd‡	Kotzebue Sound	8/23–8/28/2007	Summer movements, anomalous event	T, O	Directed movement, concentrate in very shallow water	Predator avoidance	_	A	50	F, M	A
G	Mixed-age herd†	Kasegaluk Lagoon	6/27/1988	Spring migration, staging	T, M, S	Directed movement, milling, close association, turning	Migration, social interaction	_	A, J, C	27	F, M	A, J, C
	Mixed-age herd	Kasegaluk Lagoon	7/4/1993	Spring migration, staging	T, M, S	Directed movement, milling, close association, turning	Migration, social interaction	_	A, J, C	47	F, M	A, J, C
	Mixed-age herd	Kasegaluk Lagoon	6/26/1994	Spring migration, staging	T, M, S	Directed movement, milling, close association, turning	Migration, social interaction	_	A, J, C	22	F, M	A, J, C
	Mixed-age herd	Kasegaluk Lagoon	6/30/1995	Spring migration, staging	T, M, S	Directed movement, milling, close association, turning	Migration, social interaction	_	A, J, C	18	F, M	A, J, C
	Mixed-age herd	Kasegaluk Lagoon	6/30/1996	Spring migration, staging	T, M, S	Directed movement, milling, close association, turning	Migration, social interaction	_	A, J, C	35	F, M	A, J, C
	Mixed-age herd	Kasegaluk Lagoon	6/29/1999	Spring migration, staging	T, M, S	Directed movement, milling, close association, turning	Migration, social interaction	_	A, J, C	39	F, M	A, J, C
	Mixed-age herd	Kasegaluk Lagoon	7/3/2001	Spring migration, staging	T, M, S	Directed movement, milling, close association, turning	Migration, social interaction	_	A, J, C	42	F, M	A, J, C
	Mixed-age herd	Kasegaluk Lagoon	7/7/2002	Spring migration, staging	T, M, S	Directed movement, milling, close association, turning	Migration, social interaction	_	A, J, C	50	F, M	A, J, C
	Mixed-age herd	Kasegaluk Lagoon	6/23/2003	Spring migration, staging	T, M, S	Directed movement, milling, close association, turning	Migration, social interaction	_	A, J, C	36	F, M	A, J, C
	Mixed-age herd	Kasegaluk Lagoon	6/18/2004	Spring migration, staging	T, M, S	Directed movement, milling, close association, turning	Migration, social interaction	_	A, J, C	41	F, M	
	Mixed-age herd	Kasegaluk Lagoon	6/26/2005	Spring migration, staging	T, M, S	Directed movement, milling, close association, turning	Migration, social interaction	_	A, J, C	41	F, M	
	Mixed-age herd	Kasegaluk Lagoon	7/13/2006	Spring migration, staging	T, M, S	Directed movement, milling, close association, turning	Migration, social interaction	_	A, J, C	28	F, M	
	Mixed-age herd	Kasegaluk Lagoon	6/22/2007	Spring migration, staging	T, M, S	Directed movement, milling, close association, turning	Migration, social interaction	_	A, J, C	64	F, M	
	Mixed-age herd	Husky Lakes	11/9/1989	Ice-entrapment event	T, M	Traveling, milling		_	A	9	F, M	A
	Mixed-age herd	Husky Lakes	11/17/1989	Ice-entrapment event	T, M	Traveling, milling		_	A, C	4	F, M	A
	Mixed-age herd	Husky Lakes	12/7/1996	Ice-entrapment event	T, M	Traveling, milling		_	A, J, C	17	F, M	A, J, C
	Mixed-age herd	Cook Inlet	8/28/1996	Mass-stranding event	_	Mass mortality		60	A	5	M, –	A
H	Daily aggregation	Cunningham Inlet	7/24/1998	Summer aggregation	T, S, O	Individuals biopsied from one location over 10 h	Molt	_	A, J	9	F, M	A, J
	Daily aggregation	Cunningham Inlet	7/25/1998	Summer aggregation	T, S, O	Individuals biopsied from one location over 9 h		_	A, J	9	F, M	A, J
	Daily aggregation	Cunningham Inlet	7/27/1998	Summer aggregation	T, S, O	Individuals biopsied from one location over 2 h		_	A	5	F, M	A
	Daily aggregation	Cook Inlet	10/13/1995	Resident population, aggregate at river mouth	M	Milling in same river mouth	Feeding	_	A, J	2	F, M	A, J
	Daily aggregation	Cook Inlet	10/7/1996	Resident population, aggregate at river mouth	M	Milling in same river mouth	Feeding	_	A	3	M	A
	Daily aggregation	Cook Inlet	5/13/1998	Resident population, aggregate at river mouth	M	Milling in same river mouth	Feeding	_	A	2	F, M	A
	Daily aggregation	Cook Inlet	5/31/1999	Resident population, aggregate at river mouth	M	Milling in same river mouth	Feeding	_	A, J	2	F, M	A, J
	Daily aggregation	Cook Inlet	8/29/1999	Resident population		At same location		_	A	3	F, M	A
	Daily aggregation	Cook Inlet	8/11/1999	Resident population		Sampled at same location within a few hours		_	A	2	F, M	A
	Daily aggregation	Cook Inlet	8/13/2001	Resident population		Sampled at same location within a few hours		_	A	2	F	A
	Daily aggregation	Cook Inlet	8/3/2002	Resident population		Sampled at same location within a few hours		_	A	3	F, M	A
	Daily aggregation	Anadyr Bay	8/25/2008	Summer aggregation	M	Sampled at same location within a few hours		_	A, J, C	7		
I	Multi-day aggregation	Svalbard	8/23–8/24/1998	Summer aggregation	T, M, S	Sampled at same location		_	A, J, C	10	F, M	A, J, C
	Multi-day aggregation	Svalbard	10/14–10/19/2000	Fall aggregation	T, M, S	Sampled at same location		_	A, J	6	F, M	A, J
	Multi-day aggregation	Svalbard	10/17–10/19/2001	Fall aggregation	T, M, S	Sampled at same location		_	A, J, C	8	F, M	A, J, C
	Multi-day aggregation	Anadyr Bay	8/22–8/25/2008	Summer aggregation	T, M, S	Sampled at same location		_	A, J, C	13		

Sampling details and sex determinations from the genetic analysis are also included.

* The four behavioral categories are: Travel (T), Social (S), Mill (M), and Other (O). See Appendix 1 for details

** Age categories: Adult (A): full length, white; Juvenile (J) ≥ 2/3 length-full length, grey-white; Calf (C): ≤ 2/3 length, grey

^†^Whale herds at Kasegaluk Lagoon were typically driven, sometimes for several hours, prior to arrival at the lagoon. To minimize the influence of hunting, descriptions of behaviors did not occur immediately prior, during or immediately after hunting activities

**Figure 2:**
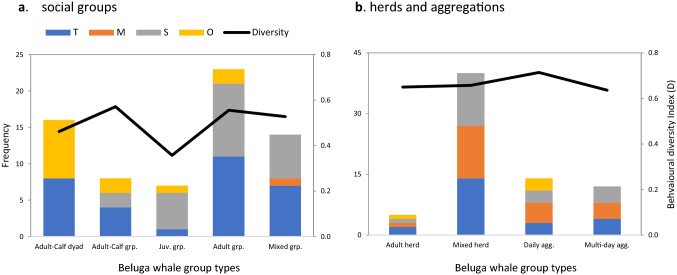
A bar chart showing the frequency and diversity of behaviours observed in beluga whale social groups and herds. Behavior is grouped into four broad categories: (T) *Travel*, (M) *Mill*, (S) *Social*, and (O) *Other*. Frequencies are indicated by stacked columns scaled to the primary *y*-axis. The behavioural diversity index, D, (see text) is indicated by a line scaled to the secondary *y*-axis. Panel (**a**) summarizes findings for beluga whale social groups, panel (**b**) for the herds and aggregations.

The most commonly observed behavioural category was *Travel* followed by *Social*, *Milling* and *Other*. It should be noted that the diversity of behaviours observed within groups was likely influenced by the amount of time the whales were under observation; in some cases, groups of whales were under observation for only a few minutes before biopsy sampling commenced, so were typically only observed travelling. Similarly, whale behaviour in some instances was clearly influenced by human activity; whales at Kasegaluk Lagoon were driven into the lagoon by hunters. Undisturbed social groups were typically observed performing behaviours that fell under a single category, primarily *Travel* or *Social*, with some recorded in the *Other* behavioural category that likely reflected molting or natal care (Fig. 2a, SI Appendix 1). Herds and large aggregations tended to conduct a wider array of behaviours, hence scoring highest D values (Fig. 2b). We found limited evidence of statistically significant differences in behaviour among groupings at the primary behavioural category level. Group type A exhibited significantly higher frequencies of *Other* behaviour than most other group types (*Fisher’s exact-test p* = 0.13–0.004) that involved very close associations between the adult and calf, suggestive of natal care (SI Appendix 1). Aggressive behaviour to non-group members, suggestive of dominance behaviour, was observed only in group type D, while interactions suggestive of likely play behaviour was observed only in group types C and E (Table 1).

### Molecular genetic analysis

PCR-based sex identification revealed that group Type E and Type G typically contained both males and females. By contrast, Type D and Type F were almost exclusively comprised of males (Table 1). The one exception was an adult-only herd observed in Kotzebue Sound, Alaska, where the sex identification assay revealed that 3 of 41 whales were female. For all Type A groups, the adults were determined to be females.

**Figure 3:**
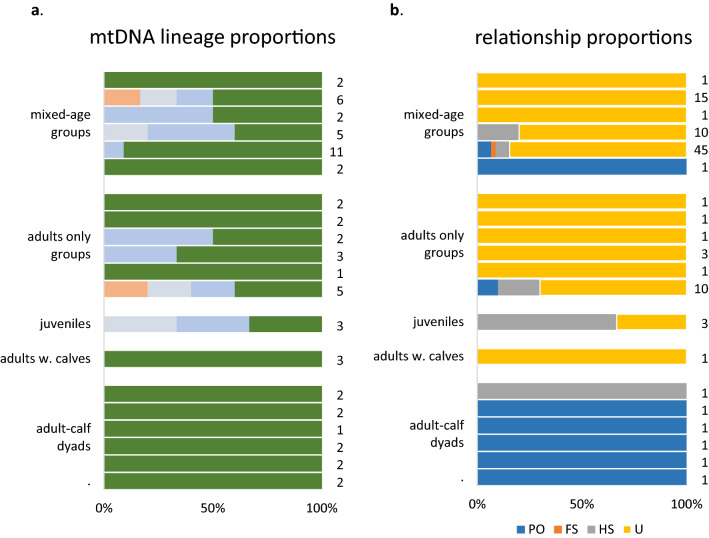
A horizontal bar chart showing the proportion of the different mtDNA lineages observed, and of the four pairwise genealogical relationships estimated, within beluga whale social groups. Each horizontal bar represents an individual social group where multiple individuals (n ≥ 2) were sampled. (**a**) Each colour represents a unique mtDNA lineage, and the number of samples successfully sequenced appear on the right; (**b**) colours represent the proportions of parent–offspring (PO), full-sib (FS), half-sib and grandparent–grandchild (HS), and unrelated (U) pairings, and the number of pairwise comparisons appears on the right. Note, in the case of the mtDNA results that the occurrence of the same colour across groups does not necessarily indicate that the same lineage was found in both.

**Figure 4:**
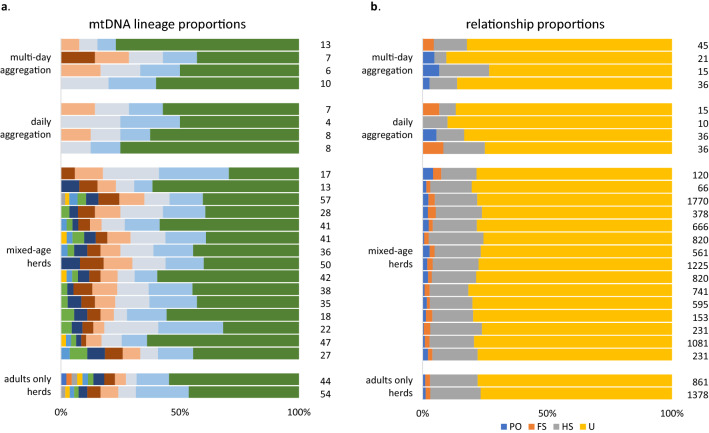
A horizontal bar chart showing the proportion of different mtDNA lineages observed and the proportion of four pairwise genealogical relationships estimated within beluga whale herds and aggregations. Each horizontal bar represents an individual herd/aggregation where multiple individuals (n ≥ 5) were sampled. (**a**) Each colour represents a unique mtDNA lineage, and the number of samples successfully sequenced appear at the right; (**b**) Colours represent the proportions of parent–offspring (PO), full-sib (FS), half-sib and grandparent–grandchild (HS), and unrelated (U) pairings, and the number of pairwise comparisons appear at the right. Note, in the case of the mtDNA results that the occurrence of the same colour across groups does not necessarily indicate that the same lineage was found in both.

Beluga whale groups were frequently composed of more than one maternal lineage (Figs. 3, 4). Other than adult–calf dyads (see below), groups of beluga whales even with as few as 2 individuals older than dependent calves were found to contain whales that had different mtDNA lineages (Fig. 3). The number of distinct mtDNA lineages found within social groups ranged from 1 to 4 (Fig. 3), while the number observed within herds and aggregations ranged from 3 to 12 (Fig. 4).

All but one of the adult–calf dyads were determined to be mother–calf dyads; the single remaining dyad did not match at one locus, and ml-relate conservatively estimated the relationship to be a half sibship (Fig. 3). However, when a genotyping error rate of 0.05 was used, a parent–offspring relationship was found to be more likely. Apart from mother–calf dyads, beluga whale groupings of almost all types contained a mixture of closely related and either distantly or unrelated individuals (Figs. 3, 4). Large herds and aggregations were particularly dominated by distantly or unrelated (U) pairings (Fig. 4b) even though they also contained first (PO) and second (FS, and HS) order relationships.

Due to statistical power considerations, the demrelate analysis was conducted only on migrating herds and seasonal aggregations where the sample size exceeded 14 individuals. The analysis revealed that for the relatedness estimator *M*_*xy*_ the observed frequencies of FS and HS in these larger groupings were significantly higher than expected frequencies of sibships in a randomly generated population with the same allele frequencies and with the same sample size (Table 2). By contrast, the observed frequencies of sibships using the *r*_*xy*_ estimator were often lower than random expectations (Table 2). However, further tests on sample sets with artificially inflated frequencies of siblings revealed that the *r*_xy_ estimator consistently overestimated expected frequencies (Supplementary Table S1 online).

**Table 2:** The observed frequencies of siblings (FS and HS) in beluga whale herds compared to random expectations using the program DEMERELATE.

	n†	run #	M_xy_			r_xy_		
			Observed*	Expected	*p* value‡	Observed	Expected	*p* value
Kasegaluk Lagoon 1993	38	1	0.354	0.225	***	0.265	0.276	NS
		2	0.333	0.220	**	0.182	0.211	NS
		3	0.333	0.211	***	0.225	0.270	**
Husky lakes 1996	14	1	0.397	0.236	*	0.192	0.346	**
		2	0.397	0.231	**	0.218	0.372	**
		3	0.167	0.128	NS	0.192	0.244	NS
Kasegaluk Lagoon 1998	42	1	0.467	0.294	***	0.265	0.309	**
		2	0.242	0.146	***	0.254	0.279	NS
		3	0.467	0.288	***	0.319	0.382	***
Kasegaluk Lagoon 2001	25	1	0.243	0.147	***	0.243	0.240	NS
		2	0.243	0.130	***	0.237	0.243	NS
		3	0.243	0.137	***	0.227	0.30	**
Kotzebue Sound 2007	23	1	0.411	0.265	***	0.182	0.237	NS
		2	0.411	0.273	***	0.178	0.253	**
		3	0.411	0.249	***	0.182	0.249	*

Two estimators of pairwise relatedness were compared, M_xy_ and r_xy_, and multiple runs were conducted for each herd. All herds yielded similar test outcomes. Results are provided for a number of the herds tested.

^†^Sample sizes are smaller than total sample size as one individual from each PO pair was excluded from the test (see text for details).

*Observed proportions may differ among runs because the calculated relatedness thresholds for FS and HS may differ among runs.

^‡^Statistical significance is denoted as follows: NS—*p* > 0.05; **0.001 < *p* ≤ 0.05, ***p ≤ 0.001.

**Figure 5:**
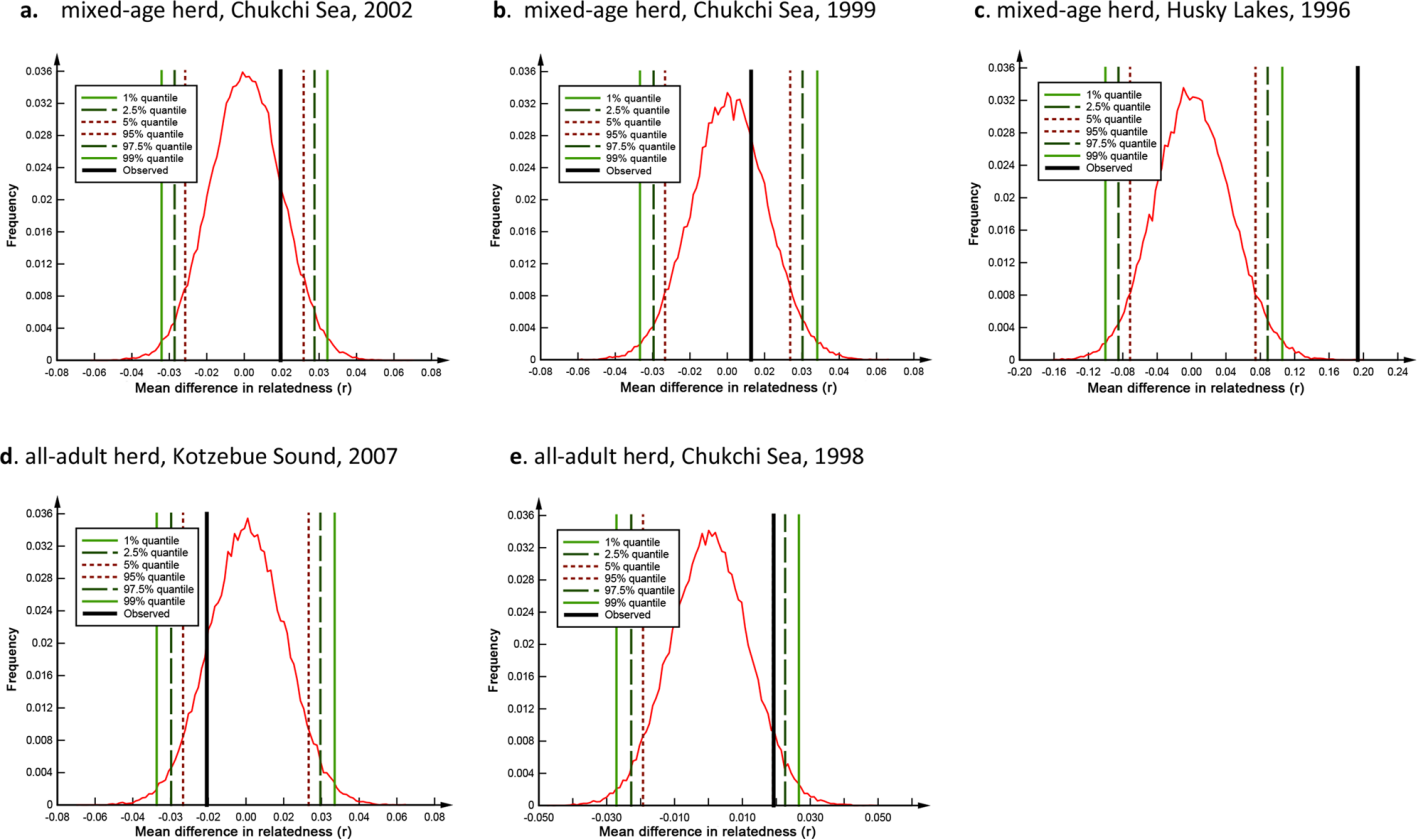
A series of graphs sumamrizing the outcomes of tests of differences in mean relatedness, *r*, within matrilines compared to mean *r* between matrilines in beluga whale groupings using COANCESTRY v. 1.01.10. Results from a subset of herds with multiple mtDNA haplotypes are shown using the moment estimator *r*_*QG*_ of Queller and Goodnight (1989). If the observed difference (black line) falls outside the 90% (dotted lines), 95% (dashed lines), and 99% (green solid lines) confidence intervals from the bootstrap analysis distribution the difference is adjudged to be significant.

While the herds and aggregations typically comprised multiple mtDNA lineages, there was no clear evidence that each lineage represented an extended matrilineal family. For most groups, average relatedness among group members (both within and between lineages) was low, ranging from $\overline{r}$ = − 0.03 to 0.04. While $\overline{r}$ within mtDNA lineages tended to be higher, this was not statistically significant in most herds analyzed (*p* > 0.05; Fig. 5). The one exception was the ice-entrapped herd involved in Husky Lakes adjacent to the Canadian Beaufort Sea ($\overline{r}$_within_ = 0.15 vs. $\overline{r}$_between_ = − 0.03, *p* < 0.01) in which several mother–calf dyads were sampled.

**Figure 6:**
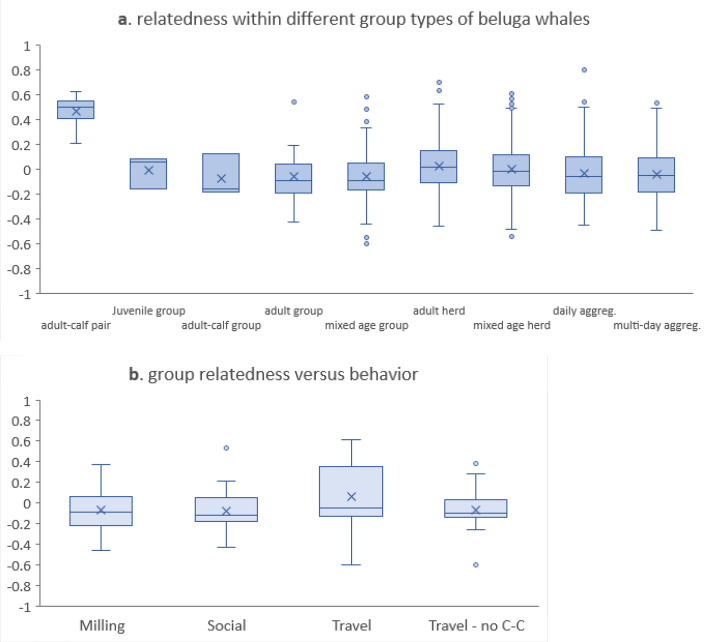
Box and whisker plots showing the patterns of relatedness within and between beluga whale group types and behaviors. The median (horizontal bar) not the mean was used as the central tendency. Boxes encompass the inter-quartile range (IQR) around the median, and the whiskers capture the range that is 1.5 times the IQR. Values outside this range were identified as outliers. (**a**) Observed pairwise *r* across group types; (**b**) observed *r* by behavioral category. Travel was assessed for all group types including adult–calf dyads (labelled ‘Travel’), as well as for all group types excluding the adult–calf dyads (labelled ‘Travel—no C–C’).

Comparing patterns of pairwise r within and between the different types of whale groups revealed that apart from adult–calf dyads, beluga whale social groups, herds, and aggregations had mean and median pairwise r values close to zero (Fig. 6a). Furthermore, we found little evidence of significant differences in $\overline{r}$ among grouping types, apart from all comparisons involving adult–calf dyads ($\overline{r}$_*QG*_ = 0.468 *p* = 0.0001) and some comparisons for all-adult herds ($\overline{r}$_*QG*_ = 0.022 *p* = 0.0001 to 0.770). Behaviour did not appear to be related to median relatedness within groups (*t*-test *p* > 0.05; Fig. 6b), although groups observed conducting *Social* behaviour had significantly lower mean relatedness ($\overline{r}$_*QG*_ = − 0.082) than those involved in *Travel* ($\overline{r}$_*QG*_ = 0.065; *p* = 0.03). This was driven primarily by the adult–calf dyads all of which were recorded as travelling (Fig. 6b).

**Figure 7:**
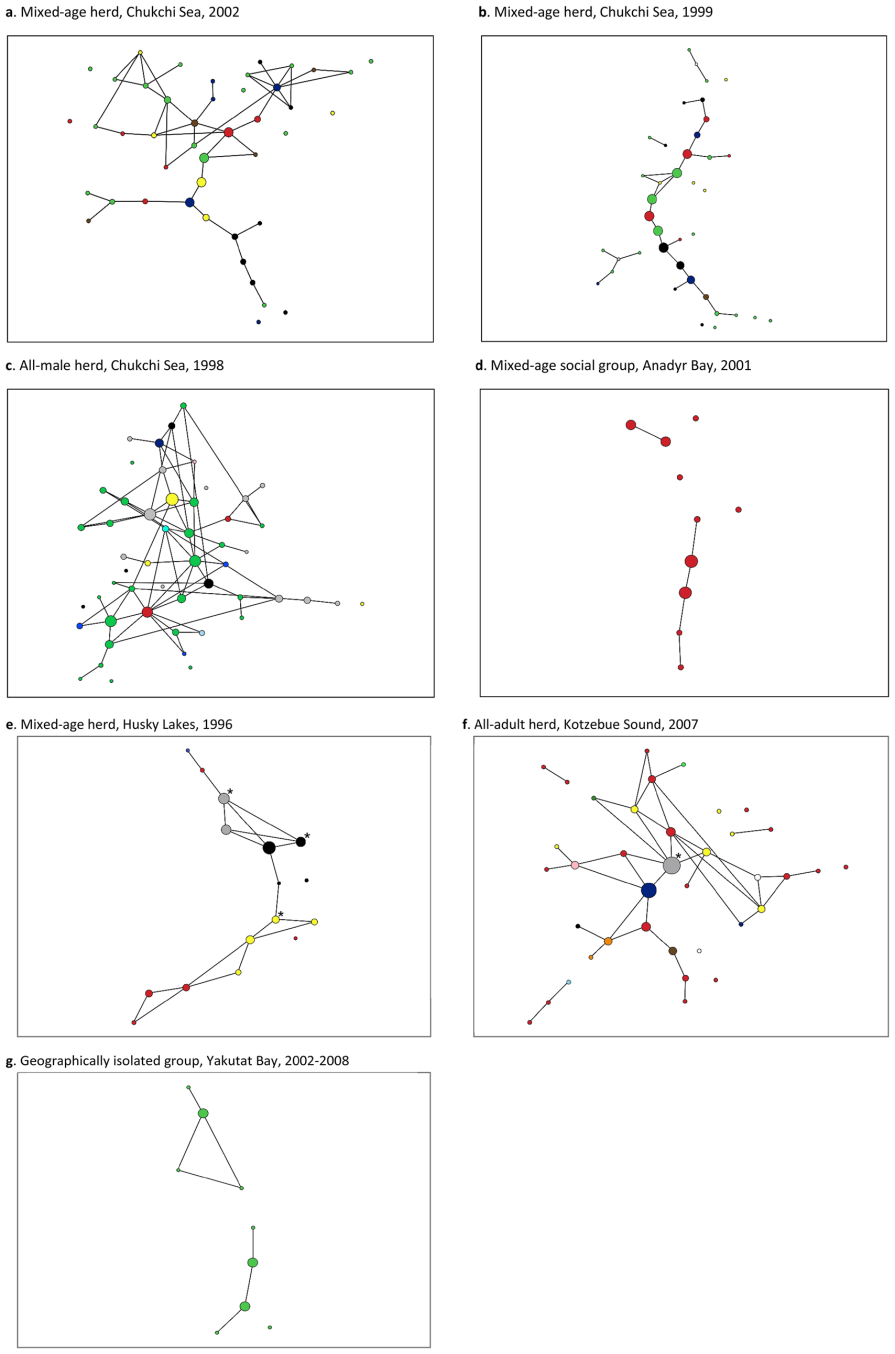
Networks of beluga whale social groups and herds based on pairwise genetic relatedness. Node size reflects betweenness centrality, node colour represents mtDNA lineage. Percolation thresholds were set to the point where links among unrelated pairs of whales were excluded. Calves in the Husky Lakes network are indicated by asterisks. The lone female in the Kotzebue Sound network is indicated by an asterisk. The Yakutat network is a compilation of the majority of individuals in a small geographically isolated group of whales (N_min _≈ 12) sampled across 7 years. Networks were constructed using EDENetworks v. 2.18.

Beluga whale networks based on genetic relatedness were characterized by long paths that connected through a few central individuals (Fig. 7). Both the automatic and manual thresholds of most networks revealed that few social groups or herds formed a “connected network”, which is a network that consists of a single component where all nodes (individuals) could reach every other node via some path (Fig. 7). Most individuals within a beluga whale social group or herd were directly linked to just one or two close relatives ($\overline{k}$ = 1.40–2.85) who in turn, were linked to a few other whales, thus forming long, interconnected paths (Fig. 7). Regularly, a smaller number of animals had links to more individuals (*k* = 3–10) while some individuals were not connected (*k* = 0) to other whales in the group or herd at all. The betweenness centrality value for individual nodes varied greatly within most networks (e.g., *bc* = 0–279), further indicating that beluga whale networks were not fully connected and generally comprised long paths that interconnected through a few individuals. The clustering coefficient of individual nodes was generally low ($\overline{C}$ = 0.06–0.5), indicating that in most cases where an individual was closely related to two or more other individuals, those other whales were not closely related to each other. There were exceptions to this, as can be seen from the highly reticulated elements of some of the networks (e.g., Fig. 7a, c). These highly connected network ‘neighbourhoods’ likely indicated multiple familial relatives.

**Figure 8:**
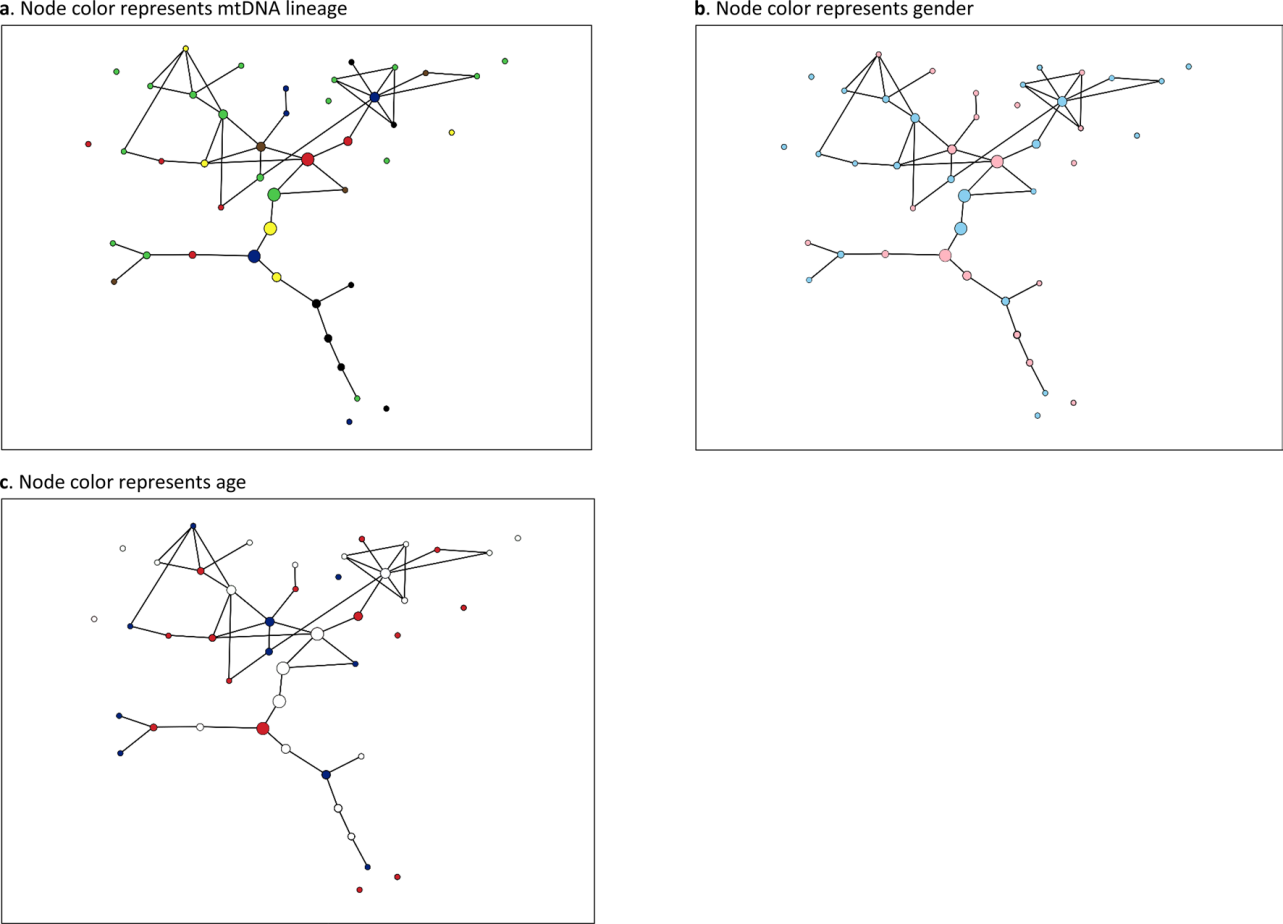
Network of a mixed-age beluga whale herd from the Chukchi Sea based on pairwise genetic relatedness. Node size reflects betweenness centrality. (**a**) node colour represents mtDNA lineage; (**b**) node colour represents sex: male (blue), and female (pink); (**c**) node colour represents age: juvenile (blue), young adult (white), old adult (red). Networks were constructed using EDENetworks v. 2.18.

There was limited evidence of structuring within the genetic networks based on maternal family lines. While some networks contained elements where a number of linked individuals had the same mtDNA haplotype, in most cases links occurred among individuals who possessed different haplotypes (Fig. 7). This was even the case in the highly connected familial neighbourhoods mentioned above indicating paternal rather than maternal relatedness. The network properties of betweenness centrality (*bc*), degree (*k*), and clustering coefficients (*C*) were rarely found to differ among haplotypes within networks (*t*-test and one-way Anova, *p* = 0.01–0.72). There was also no apparent difference between the properties of males and females in the networks (e.g., Fig. 8b). For example, *bc* did not differ significantly among the sexes (*p* = 0.14–0.8) and neither did *C* (*p* = 0.16–0.98) or *k* (*p* = 0.16–0.91).

There were a number of herds (Kasegaluk Lagoon and Husky Lakes) where age estimates were available for all individuals. Age categories did not significantly influence the location or other network properties of individuals within the network (anova, *p* = 0.08–0.76). Juvenile and subadult whales were as likely to be at the center or periphery of the network (*bc*), have as many links to other individuals (*k*), and form reticulated clusters with multiple whales that were also related to each other (*C*), as adult animals were (e.g., Fig. 8c). In the one instance where multiple calves were sampled, the calves had central positions within the network ($\overline{k}$ = 3.33) and relatively high clustering ($\overline{c}$ = 0.61), and betweenness ($\overline{bc}$ = 21.33) compared to the entire network, though not significantly so (*p* = 0.17–0.64, Fig. 7e).

Interestingly, the networks of all-male herds were quite similar to those of mixed herds (Fig. 7c). The male herds were generally comprised of only adult animals, with one exhibiting greater connectively among individual whales than all other herds sampled in that population (Fig. 7c).

**Figure 9:**
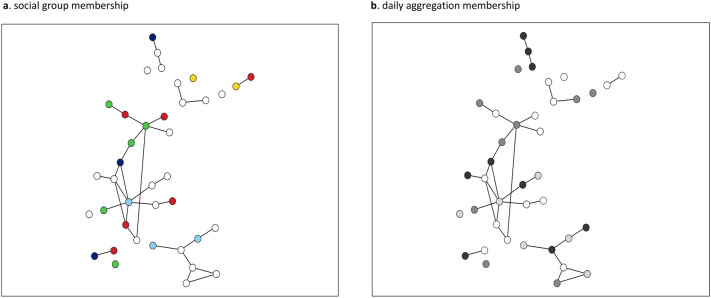
Network of the beluga whales that were biopsy sampled in Cunningham Inlet in the summer of 1998 based on pairwise genetic relatedness. Node and edge size are fixed, and percolation thresholds were set to the point where links among unrelated pairs of whales were excluded. (**a**) Nodes with the same colour indicate whales that were in the same social group when sampled. For example, the three dark blue nodes indicate three individuals from an all-juvenile group, while the six red nodes indicate individuals from a mixed-age group. (**b**) Nodes with the same shading indicate whales that were in the same daily aggregation when sampled. White coloured nodes indicate whales that were not sampled in a social group or within a daily aggregation, respectively. Networks were constructed using EDENetworks v. 2.18.

Network analysis of social groups was not very informative. However, in one location, (Cunningham Inlet, Canada), where several groups within a large summer aggregation were biopsied over a period of 5 days, individuals observed associating in a distinct group typically did not cluster together within the network (Fig. 9a). Conversely, closely related whales that were not observed associating, were often sampled nearby in the same day or on subsequent days (Fig. 9b).

In some locations whales that were observed associating were caught, satellite tagged, and subsequently tracked, providing an opportunity to assess seasonal movement and association patterns in relation to genetic relatedness. Five adult male belugas tagged from the same herd in Kasegaluk Lagoon had similar seasonal movements while the tags transmitted (n = 13–104 days), and some appeared to travel as a group for periods of up to 29 days^43^. Interestingly, none of these whales were closely related. In Svalbard, three adult male whales travelling together were tagged at the same time and they moved together throughout the subsequent months (for as long as the tags transmitted—n = 52–120 days)^44^. Again, they were not closely related. Finally, three young adult males that were tagged at another location in Svalbard, this time over three successive days spent most of the time together, occasionally splitting up only to come back together, over the timeframe that the tags transmitted (n = 10–63 days) (Lydersen personal communication); the individuals were not closely related.

## Discussion

Beluga whales formed a variety of group types that were consistently observed in multiple populations across the species range and certain behaviours were associated with grouping type (Table 3). Similar grouping patterns have been observed by others^16,21–23,45–47^ and a diverse range of behaviours have also been described for beluga whales in the wild. A number of these behaviours have been linked to specific vocalizations^20,48,49^, have been found to be influenced by environmental conditions and spatiotemporal variables^21,50–52^, or found more commonly associated with specific group types^21,41,49^. However, until now there have been limited formal analyses of the relationship between behaviourss, group type, group dynamics, and kinship.

**Table 3:** Beluga whale group types, their behavior and likely function, and the degree to which they are kin-based.

	Type of group	Proportion comprised of close kin	Composition	Behaviors and likely function	Original field description	Similar group types and behaviors in the literature
A	Mother–calf dyad	High	Mother–calf pair	All three mother–calf group types engage in behaviors likely related to natal care and possibly shared care of young	Adult–calf dyad	^19,21,41^
	Mother–calf triad	–	Likely mother with two calves		Triad	
B	Mother–calf group	Intermediate	Likely coalescence of mother–calf dyads/triads, not all group members are close relatives		Adult–calf group	
C	Juvenile-only group	Low	Calves of multiple ages and different maternal lineages	Behaviors likely indicate play and possibly reciprocal social learning	Juvenile-only group	^19,41^
D	Adult-male group	Low	Typically, between 2 and 15 whales; multiple maternal lineages	Coordinated behaviors suggestive of socio-sexual dominance, male reproduction	Adults-only group	^21,23^
E	Mixed-age group	Low	Typically, less than 10 individuals, of both sexes and all ages; multiple maternal lineages	Diverse behaviors that indicate social interaction, likely feeding, and travel	Mixed-age group	^23^
F	Adult-male herds	Low	> 50 to several hundred individuals; multiple maternal lineages	Diverse behaviors that indicate social interaction, predator avoidance, likely feeding and migration	Adult-only herd	^45,46^
G	Mixed-age herds	Low	> 50 to low thousands of individuals, of both sexes and all ages; multiple maternal lineages; limited evidence of preferential associations among maternal kin	Diverse behaviors including social interaction, feeding, and migration; dynamic internal grouping patterns	Mixed-age herd	^16,45,46^

A more detailed summary of beluga whale grouping types can be found in Supplementary Information.

Our genetic analysis revealed several unexpected results. Beluga whale groupings (beyond mother–calf dyads) were not usually organized around close maternal relatives. The smaller social groups, as well as the larger herds, routinely comprised multiple matrilines. Even where group members shared the same mtDNA lineage, microsatellite analysis often revealed that they were not closely related (but see Husky Lakes), and many genealogical links among group members involved paternal rather than maternal relatives (Figs. 3, 4, 7). These results differ from earlier predictions that belugas have a matrilineal social system of closely associating female relatives^21,24^. They also differ from the association behavior of the larger toothed whales that informed those predictions. In ‘resident’ killer whales, for example, both males and females form groups with close maternal kin where they remain for their entire lives^25,28,53^. Both long-finned (*Globicephala melas*) and short-finned (*G. macrorhynchus*) pilot whale societies are structured along similar lines^26,54^, while female sperm whales (*Physeter macrocephalus*) form stable multi-generational matrilineal social units^27,29^.

In several cases males held central positions within reticulated ‘neighbourhoods’ of networks, indicating that they were closely related to multiple group members, some of whom were likely their offspring or grandoffspring (Fig. 8). The occurrence of paternal relatives within the same grouping was even more evident in the all-male herds (Fig. 7c, f). These findings indicate that male belugas may exhibit high fidelity to a herd for much of their lives, often associating with adult offspring of both sexes. Furthermore, males may be highly philopatric to their natal herd and thus associate with parents and grandparents.

The brief periods of observation of most beluga groups in this study combined with the much longer periods tracking satellite tagged whales^43,44^ provided important insights into the stability and dynamics of grouping patterns. Close relatives did not always associate in a group, but the fact that they could be in another group close by was supported by field observations where individually recognized whales were observed moving between groups, and even group types, over a few days, and in some cases a few hours (O’Corry-Crowe, field notes). By contrast, unrelated whales can spend long periods of time and cover considerable distances together, and sometimes split up only to come back together.

The relationships we found between group type, behaviour, dynamics and kinship indicate that the driving forces behind social structure in beluga whales are complex. Grouping patterns may depend on the social context (i.e., who is present), as has been proposed for bottlenose dolphin fission–fusion societies^30^, but also on life history and the behavioural/ecological context including: breeding, migration, feeding, vigilance and natal care. To what degree these groupings are cooperative or selfish is not clear. More remains to be learned about the longevity, stability and kin composition of beluga groupings before clear hypotheses about inclusive-fitness benefits versus non-kin-based advantages of group membership can be tested. Recently, female kinship has been identified as central to social complexity in cetacean species with kin selection as a primary evolutionary driver of cooperation, life history and culture^55^. However, there are already indications that more than one evolutionary mechanism may be involved in beluga whales. For example, the small groups comprising two or three large males may be similar to male alliances in bottlenose dolphins^30,56^ or coalitions in lions (*Panthera leo*)^57,58^ and chimpanzees (*Pan troglodytes*)^59^, where group members cooperate primarily to secure reproductive benefit. If these beluga affiliations are cooperative in nature, the finding that group members tended to be unrelated (Fig. 3) indicates that direct fitness benefits in terms of improved reproductive success (and possibly survival) to group members may be garnered via reciprocity^2,3^, mutualism^4^ and/or manipulation^5^.

Several genetic studies have revealed a strong tendency for beluga whales to remain in their natal subpopulation or population^35–40^. When viewed with the current study’s findings this provides more insight into the social, ecological and demographic scales at which beluga whale societies may be operating. We propose that beluga whales, across a wide variety of habitats and among both migratory and resident populations, form communities of individuals of all ages and both sexes that regularly number in the hundreds and possibly the thousands. Beluga whales may form a wide variety of social groupings within these communities, dependent on immediate social and ecological contexts, that may include seasonal sexual segregation. At larger spatiotemporal scales there is strong philopatry or fidelity by both sexes to these mixed-age and—sex communities.

We have shown that beluga whale societies pose several challenges to emerging explanations for the evolution of sociality, culture and unique life history traits in toothed whales. The stable matrilineal societies of killer, sperm, and pilot whales seem to fit neatly with the theory of kin selection, anchored in inclusive fitness benefits gained by associating and cooperating with close relatives^55^. Inclusive fitness also underpins evolutionary explanations for a rare phenomenon in nature: menopause, which has only been recorded as prevalent in a few vertebrate species that includes humans and four toothed whale species (including beluga whales)^60–62^. New research on killer whales, for example, found that assistance provided by post-reproductive grandmothers improved the survival of their grandoffspring^63^. Some of these matrilineal whales may form matriarchal societies where older females have substantial influence over kin as seen in other, long-lived matrilineal species including African elephants (*Loxodonta africana*)^61,64–66^. Stable, multi-generational matrilineal whale societies also seem ideal environments for the emergence of cultures because of the inclusive fitness benefits of transmitting behavioural traditions and ecological knowledge to close kin^13^, which in turn may influence gene evolution and even speciation^67,68^.

On the face of it, beluga whales seemed to fit this model; they form multi-generational groupings^16,21,23^, females have long post-reproductive lifespans^46,62^, and the prolonged period of maternal care seems the likely conduit for social learning and the emergence of migratory culture^40^. Our study did find that close kin, including close maternal kin, regularly interact and associate. However, it also revealed that beluga whales frequently associate and interact with more distantly related and unrelated individuals. Inclusive fitness benefits alone seem insufficient explanations for the evolution of group living in beluga whales. The frequency with which adult female belugas associate, and presumably cooperate, with non-kin also complicates studies of menopause where contributing to the fitness of kin (along with a long lifespan) is considered the basis for its evolution^61,63,69,70^. Our findings indicate that evolutionary explanations for group living and cooperation in beluga whales must expand beyond strict inclusive fitness arguments to include other evolutionary mechanisms.

Belugas likely form multi-scale societies from mother–calf dyads to entire communities. The longevity and stability of a grouping, and the adaptive advantages to the individual of being a group member, likely differs at these different scales. While membership in social groups can be highly dynamic, both males and females appear to be highly faithful to their community. These behaviours in concert with a long lifespan (≥ 70 years)^46^ create an environment where frequent interactions may occur, and long-term relationships may develop, among both kin and non-kin of differing ages and both sexes. In such a social setting inclusive fitness benefits, such as the care of non-descendent young and group leadership, may maintain cooperation among close kin (kin selection). Similarly, non-kin could benefit directly from the vigilance of others or indirectly from the sharing of ecological knowledge (mutualism). For example, beluga whales have been observed to rapidly respond *en masse* to the presence of a predator, including dispersing from a killer whale attack site for several days^71^ and actively encircling and encroaching on a polar bear until it swam ahore^72^. Furthermore, frequent interactions among non-kin over a long life may provide ample opportunities for receiving delayed benefits from cooperative exchanges (reciprocity). The prevalence of reciprocity in animal societies, however, is widely debated^5,73^ and would be challenging to study in wild belugas. Unlike the matrilineal whales, perhaps, the regular occurrence of adult males as well as females in mixed migrating herds and summer aggregations, when taken with evidence of both male and female philopatry^35–40^ suggests that elders of both sexes may be important repositories of social and ecological knowledge in belugas. In these communities social learning may occur among non-kin as well as kin, facilitating the emergence of cultures (e.g., the development and perpetuation of migratory circuits and the use of traditional feeding areas) that are beneficial to all members of the community. Similar arguments may apply to the evolution of social structure in other cetacean species that form fission–fusion societies and/or are non-matrilineal and where groups comprise both kin and non-kin including bottlenose dolphins^30^, northern bottlenose whales (*Hyperoodon ampullatus*)^74^ and possibly Baird’s beaked whale (*Berardius bairdii*)^75^.

From these perspectives, beluga communities have similarities to human societies where social networks, support structures, cooperation and cultures involve interactions between kin and non-kin^76,77^. An analysis of human societies may also be instructive in understanding menopause in beluga whales. Others have argued that menopause can only evolve when inclusive fitness benefits outweigh the costs of halting reproduction early^61^. However, unlike killer and pilot whales, but like some human societies^76^, beluga whales do not solely (or even primarily) interact and associate with close kin. Nevertheless, it may be that their highly developed vocal communication^19,42^ enables beluga whales to remain in regular acoustic contact with close relatives even when not associating together, and that over the course of a long life, individual belugas preferentially assist close kin when they do encounter them, and in the case of older post-reproductive females preferentially assist maternal kin.

Matrilineal societies pose unique challenges for species management where social and cultural disruption due to the loss of adult females may have far reaching consequences to populations beyond the immediate impacts of lowered productivity^78,79^. In beluga whale societies this may also hold true, but, in addition, mortality of older males as well as older females may also increase the risk of losing important ecological and social knowledge. Furthermore, cultural conservatism may slow the recolonization of areas formerly occupied by beluga whales, may increase species vulnerability to localized threats (e.g., decline of a preferred food source), and slow behavioural and ecological adaptation to ecosystem change.

This study provides new insights into the fundamental nature of beluga whale social structure and challenges prevailing hypotheses about social organization, kinship and the selective advantages of group living in this species. A more detailed exploration of many of the study’s findings can be found in Supplementary Information online. Future research should focus on competition, conflict and selfish vs cooperative behaviour in beluga whale societies, expand genetic studies to identify more distant (≥ 3rd-order) relatives, and investigate the mechanism and the significance of the spread of cultural innovations, especially as related to population responses to climate change.

## Methods

Data on the size and composition of beluga whale groups were collected at several locations across the species range (Fig. 1). Data on behaviour were collected at a number of the locations using focal-follow sampling or opportunistic observations of animals conducted from shore, small boats or an observation tower prior to tissue sampling. Observations typically lasted from several minutes to a few hours. Detailed information on whale behaviour was recorded and classified into 4 broad categories: *Travel*, *Mill*, *Social*, and *Other* based on O’Corry-Crowe et al.^80^ (see SI Appendix 1 for details), and an index, D, was developed to quantify the diversity of behaviours recorded within beluga whale groupings: D = 1 − ((∑(d_i_^2^)(n))/n − 1), where d is the proportion of *i*th behavioural category observed and n is the number of times the behaviour was observed. At a number of locations, the data collected on group size, composition and behaviour were more limited. In some of these locations (Table 1) caution is required in interpreting field data on group characteristics because whale behaviour was likely influenced by human activities (i.e., hunting) at the time of observation.

Animals were in a group of some description if they were aggregated in space (i.e., non-uniformly distributed) at the time of observation. A distinction was made between large, loose groupings of whales, termed ‘herds’, and smaller, more compact groupings of individuals termed ‘social groups’. The former comprised groups of over 50, and as many as fifteen hundred, loosely associated animals seen in bays, inlets or estuaries. Social groups comprised between 2 and 50 whales in close association, (defined as within 12 m or up to 4 body lengths of other group members), in which physical contact between animals was common. Although the distinction between social group and herd is based on our field observations of whale behaviour and group size, the size cutoff is somewhat subjective. Similar sizes have been reported for our definition of a social group by others^16,19,21^. The smaller social groups often occurred within the larger herds. In some instances, longer-term temporal patterns of grouping behaviour were also available from a series of satellite-linked telemetry studies of beluga whale movements and dive behaviour^43,44,81,82^ that spanned periods from a few days to several months.

Tissue samples were collected from: (a) free-swimming beluga whales via remote biopsy, (b) temporarily captured whales during tagging operations, or (c) harvested whales during biological sample collection between 1988 and 2008. Details on tissue collection and preservation methods can be found elsewhere^35,83,84^. Total DNA was extracted from each tissue sample by established protocols and screened for variation within 410 bp of the mtDNA control region and eight independent microsatellite loci (see^35,40,85^ for methodological details). The sex of each sample was determined by PCR-based methods^86^, and replicate genotyping, sequencing and sex determination was conducted to estimate error rates.

Earlier studies revealed that the eight hypervariable microsatellite loci were highly informative in determining individual identity, assessing gene flow and population structure, and estimating first and second-order relationships in beluga whales^40,84,85,87^. These earlier studies also determined minimum allowable thresholds for missing data and genotyping errors and found that individual identity required a minimum of four of these loci^87^ while accurate relatedness (*r*) estimation required a minimum of six loci genotyped per individual^40^. In the current study, we continued such tests by conducting analyses on datasets with individuals scored at ≥ six loci, ≥ seven loci, and all 8 nuclear loci. With data from known cow–calf pairs, these analyses showed that individuals scored at a minimum of 6 loci provided reliable estimates of high relatedness and close genealogical relationship. In the network analysis (see below), we decided to raise this threshold to ≥ seven loci in order to avoid possible spurious network edges due to lower confidence in very low levels of estimated relatedness.

The programmes coancestry^88^ and ml-relate^89^ were used to estimate *r* and genealogical relationship among individuals based on the microsatellite data. Coancestry implements 7 estimators of *r* that use multilocus genotype data. This programme uses simulations of genotypic data of pairs of individuals with one of four predefined relationships: parent–offspring (PO), full-sib (FS), half-sib and grandchild–grandparent (HS), and unrelated (U), to determine which estimator is the best for a particular study. Using known population allele frequencies to simulate sets of paired genotypes that fit all four relationship categories (i.e., PO, FS, HS and U), which were similar in sample size to our larger group sample sets (n = 20–40), we found that of the seven *r* indices compared, the dyadic likelihood estimator, *r*_*wang*_^90^, and the moment estimator, *r*_*QG*_^91^, performed best and thus these two estimators are presented here. ml-relate, which uses a maximum likelihood approach to estimate the likely relationship between pairs of individuals for the same four relationship categories: PO, FS, HS and U, facilitated comparisons of *r* and relationships for each pair of individuals.

coancestry was used to test for differences in average *r* among groupings. Specifically, differences in mean *r* among subgroups of individuals that had the same versus different maternal lineages (i.e., mtDNA haplotypes) were tested to determine whether larger aggregations of beluga whales were composed of matrifocal family units. The observed differences were compared to a distribution of differences based on 50,000 randomized bootstrap runs of the data. Statistical hypothesis tests and descriptive statistics summarizing the patterns of *r* within different groupings were conducted in Excel 2016. The combined mtDNA-microsatellite analyses also allowed inferences of paternal relatedness when high *r* and close genealogical relationships (PO, HS, and HS) were estimated among individuals with different mtDNA haplotypes.

Because large groups of animals (e.g., herds, flocks) will likely contain a certain proportion of close relatives without necessarily indicating behavioural preferences for associations among close kin the R package demerelate v. 0.9-3^92,93^ was used to investigate whether beluga whale herds and seasonal aggregations had more close relatives than would be expected by chance. The programme was designed to assess FS, HS and U frequencies, but not PO frequencies (Kraemer personal communication). Therefore, in order to exclude PO pairs from the empirical datasets prior to running demerelate, ml-relate was used to independently estimate relationship categories (i.e., FS, HS, U and PO: see above), and then one individual from each PO pair was excluded. In the demerelate analysis, two estimators of pairwise relatedness were compared, the genotype-sharing *M*_*xy*_^94^ as recommended by the programme’s authors and the widely used *r*_*xy*_ (^91^; note this is the same as *r*_*QG*_ in coancestry). For each estimator, populations of randomized offspring and un-related individuals are generated from a reference population in order to calculate threshold values for FS, HS and U relationships. The reference population in each case was the source population for the particular beluga group. χ^2^ tests were then used to compare observed FS and HS frequencies to expected frequencies among a number of individuals (of the same sample size as the observed data) that were randomly generated from the allele frequencies of the reference population.

Network analysis was used to investigate the patterns of genetic relationships among all the individuals sampled within a social group or herd. Using the programme EDENetworks v. 2.18^95^, we built networks based on estimates of individual pairwise relatedness that were converted into genetic distance matrices. Networks were then compared based on the two estimators of *r* that performed best for our investigation (*r*_*wang*_ and *r*_*QG*_, see above) and automatic thresholding was used to identify the point at which further removal of links (termed edges) fragmented the network into small components. This typically incorporated the majority of close relationships (PO, FS, and HS) within the social group/herd into the network. These thresholds were then manually adjusted to the point where links between unrelated (U) individuals (termed nodes) were excluded. ml-relate analysis (see above) was used to identify the four relationship categories. The analysis provided descriptors of overall network topology and information on the network properties of individual whales, including: (1) *degree *(*k*)—the number of edges connected to a node; (2) *betweenness centrality *(*bc*)—the number of shortest paths running through that node; and (3) *clustering coefficient *(*C*)—the ratio of the existing number of connections between a node’s neighbours to the maximum number possible. This programme also enabled us to investigate the genetic networks in terms of other properties of the individuals within those networks, including their age, sex and maternal lineage (i.e., mtDNA haplotype).

All activities involving live whales were permitted (USMMPA #782-1719-06, NARA #2013/36156-2, GOS #2013/00050-42 a.512, NOAA782-1438) and approved by the relevant authorities in each country: the US National Marine Fisheries Service Office of Protected Resources, the Russian Federation Marine Mammal Permits Office, the Department of Fisheries and Oceans, Canada scientific licenses, and the Norwegian Animal Care Board. All activities were performed in accordance with these guidelines and regulations.

### Statement on the study of live animals

All activities involving the sampling of life animals were carried out in accordance with relevant guidelines and regulations (see “Methods” for details).

## Supplementary information

Supplementary information

Supplementary information2

## Data Availability

All data will be made available through our University (FAU) website https://pbbegenetics.wixsite.com/pbbe/. DNA sequence data is archived on GenBank (https://www.ncbi.nlm.nih.gov/genbank) and all genetic data will also be archived on the Dryad Digital repository (https://datadryad.org/stash).
