# Supplementary material for: Group structure and kinship in beluga whale societies
Source: Sci Rep. 2020 Jul 10;10:11462. doi: 10.1038/s41598-020-67314-w (PMC7351962; doi:10.1038/s41598-020-67314-w)
Supplement: Supplementary file 1 — Supplementary information [file 41598_2020_67314_MOESM1_ESM.docx]

**Group structure and kinship in beluga whale societies**

Greg O’Corry-Crowe, Robert Suydam, Lori Quakenbush, Thomas G. Smith, Christian Lydersen, Kit M. Kovacs, Jack Orr, Lois Harwood, Dennis Litovka, Tatiana Ferrer

Appendix 1: Ethogram of beluga whale behaviors at several locations across their range.

| Behavioral Category | Detailed description of behavior | Interpretation of likely function |
| --- | --- | --- |
|  |  |  |
| Travel | directed movement at or near surface, regular surfacing | travel |
|  |  |  |
| Social | contact with other whales, sharp and/or rapid turning, upside down swimming when in close association, often with audible vocalizations and bubble-blasts. Sometimes occurs in adult-calf groups | social interactions, natal care-creshe |
|  | aerial behaviors: spy hop, tail waive, tail slap | surveillance, social interactions |
|  | large adults briefly chase smaller animals, with smaller animals taking evasive action | aggression, social dominance |
|  | juveniles, occasionally with older whales, engage in contact, chase, body twisting and turning, audible vocalization, with no permanent evasive responses | social interactions, play |
|  | coordinated parallel swimming by large adults | socio-sexual behavior |
|  | lateral swimming and ventrum presentation | social-sexual behavior |
| Mill | regular surfacing and diving in same location, often close to other animals conducting the same behavior | Possible feeding |
|  |  |  |
| Other | rubbing body on substrate  very close association between calf and an adult later determined to be its mother* | molting  natal care |

*close associations between a mother and calf may also qualify as Social behavior. We chose to categorize it as Other behavior as it connotes an obligate physiological dependency.
