# Supplementary material for: Group structure and kinship in beluga whale societies
Source: Sci Rep. 2020 Jul 10;10:11462. doi: 10.1038/s41598-020-67314-w (PMC7351962; doi:10.1038/s41598-020-67314-w)
Supplement: Supplementary file 2 — Supplementary information2 [file 41598_2020_67314_MOESM2_ESM.docx]

**Group structure and kinship in beluga whale societies**

Greg O’Corry-Crowe, Robert Suydam, Lori Quakenbush, Thomas G. Smith, Christian Lydersen, Kit M. Kovacs, Jack Orr, Lois Harwood, Dennis Litovka, Tatiana Ferrer

**Supplementary Information**

**Discussion**

*The limitations of the r_xy_ estimator when comparing observed vs expected frequencies of siblings (FS and HS) in the program DEMERELATE*

In the analysis of observed FS and HS frequencies in beluga whale herds relative to expected frequencies of sibships in a randomly generated population (with the same alleles frequencies and with the same sample sizes) the study found that for r_xy_^1^ the observed frequencies of sibships rarely differed from, and in many cases were lower than, random expectations. This was surprising and differed from the allele estimator, M_xy_^2^ (Table 2), which is recommended by the program's authors.

To investigate whether these findings reflect the true nature of beluga whale herds (i.e., that they comprise less close relatives than would be expected by chance) or some intrinsic limitation of the r_xy_ estimator in DEMERELATE^3^, we ran a further series of tests on sample sets with artificially inflated frequencies of 'siblings'. To do this, we included PO pairs which equate to FS pairs in the analysis, in effect inflating the frequency of observed siblings. Despite this, the r_xy_ estimator still estimated higher frequencies of siblings expected by chance (see table below). This markedly contrasted with the findings for M_xy_ which found that observed frequencies of siblings were significantly higher than random expectations.

**Table S1**. Testing the limitations of the r_xy_ estimator when comparing observed vs expected frequencies of siblings (FS and HS) in the program DEMERELATE

|  |  |  | M_xy_ | | |  | r_xy_ | | |
| --- | --- | --- | --- | --- | --- | --- | --- | --- | --- |
|  | n† | run # | observed | expected | *p* value‡ |  | observed | expected | *p* value |
|  |  |  |  |  |  |  |  |  |  |
| Husky Lakes 1996 | 17 | 1 | 0.425 | 0.225 | *** |  | 0.183 | 0.208 | NS |
|  |  | 2 | 0.425 | 0.233 | ** |  | 0.192 | 0.325 | ** |
|  |  | 3 | 0.425 | 0.317 | NS |  | 0.217 | 0.325 | NS |
|  |  |  |  |  |  |  |  |  |  |
| Kasegaluk Lagoon 1993 | 38 | 1 | 0.387 | 0.3 | *** |  | 0.286 | 0.327 | NS |
|  |  | 2 | 0.387 | 0.259 | *** |  | 0.208 | 0.232 | NS |
|  |  | 3 | 0.206 | 0.092 | *** |  | 0.239 | 0.29 | ** |
|  |  |  |  |  |  |  |  |  |  |
| Yakutat Bay | 9 | 1 | 0.389 | 0.139 | ** |  | 0.361 | 0.417 | NS |

In discussions with the primary author of DEMRELATE, it appears that r_xy_ may be adversely influenced by low allele numbers of some loci in some datasets, and it was recommended that this estimator not be used in such tests (P. Kraemer, pers.s comm.).

*Beluga whale grouping types: a companion to Table 3. (This section could be placed in a Box in the main text)*

The basic unit of beluga societies appears to be the ***mother-calf dyad*** (Type A). This may extend at times to a ***mother-calf* *triad*** of a mother and two calves of different age. It should be noted, however, that we had only one clear example of such a grouping and were unable to sample it, and so decided to refrain from identifying such a grouping as a distinct group type until we gather further evidence. However, others have observed triads of adult females with young of different ages (e.g.,^4,5^). Mother-calf dyads and triads may coalesce to form distinct ***mother-calf groups*** (Type B). Travel and very tight associations between mother and calf were the predominant behaviours we observed in all these adult-calf groupings, followed by social interactions. We proffer that these behaviours reflect natal care and possibly shared care of young. Smith et al.^4^ speculated that older greys in triads in the Canadian Arctic may assist in the care of the younger calf while Krasnova et al.^5^ recorded adult females and juveniles in the White Sea interacting with newborn calves while the newborn’s mother rested which they interpret as likely evidence of younger whales acquiring experience in maternal care. From the limited genetic evidence it is clear that at least some group members may not be closely related. Much remains to be learned about these adult-calf groupings, including their stability and the degree of maternal relatedness, before clear hypotheses regarding inclusive fitness and non-kin-based advantages of group membership can be tested.

Another distinct grouping type is the ***juvenile-only group*** (Type C). Group members were typically observed involved in a wide range of what we collectively termed social behaviours the exuberance of which (Appendix 1) suggest play and possibly reciprocal social learning. Such groupings may comprise unrelated individuals, arise in close proximity to adult-calf groupings (Types A and B), and are clearly ephemeral with group members often observed in adult-calf groupings (see above) and mixed-age groupings (see below) shortly before or after the formation or disintegration of juvenile-only groups (O’Corry-Crowe, field notes). Krasnova et al.^5^ frequently observed similar behaviours, interpreted as play, among groups of calves in beluga whale aggregations in the White Sea in summer.

Mature male belugas may form ***adult-male groups*** (Type D) of between two and fifteen individuals, potentially more. These groupings were observed involved in coordinated behaviours that included aggression to other whales and possible ritualized behaviours that suggest social and/or sexual dominance that are likely related to male reproductive strategies (see below). Frequently, these adult-male groups comprised only two or three large males. Such pairs and trios may be similar to cooperative male alliances in dolphins (*Tursiops* sp.)^6,7^ or coalitions in lions (*Panthera leo*)^8,9^ where group members cooperate primarily to secure reproductive benefit. If these social affiliations are cooperative in nature, the finding that group members tended to be unrelated (Fig. 3) all but eliminates a role for kin-selection in their evolution. Rather, direct fitness benefits in terms of improved reproductive success and/or survival to group members may be garnered via mutualism^10^ or reciprocity^11^. The larger adult-male groups may indicate a hierarchy of alliance coalescence in beluga whales as has been described for bottlenose dolphins in Shark Bay, Australia^6,7^, or perhaps looser ‘bachelor’ groupings that may emerge when other age and sex classes are engaged in other activities, for example: natal care and play (see above).

We established through the genetic analysis that the ***mixed-age social groups*** (Type E) often comprised both males and females and while travel was the predominant behavioural category recorded social behaviours were frequently observed, some suggesting play others possible socio-sexual behaviour (Table 1, Appendix 1). Of the five social group types we recognized, Type E is perhaps the most diverse in its composition and behaviour, and thus is the most challenging to define and interpret. Mixed-age social groups are quite small, with most containing less than 10 individuals. They contain multiple maternal lineages (the only exceptions coming from Yakutat Bay which is fixed for one mtDNA haplotype^12^), and most individuals are not closely related (Fig. 3). The current evidence indicates, therefore, that these groups are predominantly non kin-based although the adaptive advantages of joining such groups are not clear. Future research should focus on the stability, dynamics and role of these groupings and whether adult male group members exhibit behaviours that do not directly involve securing access to females.

The larger herds were typically observed when beluga whales were on migration or at aggregation areas and revealed a higher diversity of behaviours than the social groups. The ***mixed-age herds*** (Type F) were also found to be mixed-sex, and often spent considerable time involved in milling behaviour. The function of such behaviour was not always apparent; it possibly involved feeding although in the case of ice-entrapped herds it was likely related to breathing in a small area of open water. Studies of population subdivision in beluga whales have tended to focus on sampling mixed-age herds (e.g.,^13^ deMarsh and Postma). Significant geographic differentiation at mtDNA and, in some cases, nDNA markers, as well as the detection of substantial numbers of close relatives migrating together^14^ and occurring at summering areas years and even decades apart^15^ reveal a strong tendency for beluga whales not to disperse from their natal subpopulation or population. From the current study’s perspectives these findings may, in fact, indicate strong philopatry to natal mixed-age herd, or perhaps a group of herds depending on how loosely we define this grouping type. All the smaller social groups likely occur within these larger herds. Thus, there appears to be a gradation of philopatry with grouping type from herds to (at least some) social groups. This may also reflect differences in the stability of group membership; beluga whales may be life-long members of their natal herd but not of some of the social groups they join. This in turn is likely influenced by the permanence versus ephemeral nature of the grouping; herds may last years, even generations, some social groups may be much shorter lived.

That the adults-only herds were comprised almost exclusively of males was a striking finding, especially as such herds may be quite large. This required a revision of our definition to ***adult-male herds*** (Type G). The Chukchi Sea herd, for example, was estimated to number several hundred. That one herd was observed during spring migration indicates that adult male belugas may migrate separately *en masse*. That the other herd was observed in summer indicates that adult males may also forage as a herd during the open water season. Taken together, these findings indicate that adult male belugas can spend considerable time both in mixed-age and gender herds and in separate all-male herds. The likely function(s) of Type G groupings is different to the Type D groupings (see above).

*Exceptions to the pattern of high mtDNA diversity within beluga groupings*

We did find exceptions to the general pattern of high mtDNA diversity within beluga whale groupings and low relatedness among individuals with the same maternal lineage. In two cases, this may be primarily a consequence of demographic history. All the sampled members in a mixed-age social group from Anadyr Bay possessed the same mtDNA haplotype (Fig 7D). However, this haplotype was the most common haplotype recorded in that population^15^ and some group members were clearly not closely related (Fig 7D). This indicates that there may be instances where more resolving power is required in the mtDNA analysis to distinguish close maternal relatives from individuals with more distant common maternal ancestry. Similarly, all the whales sampled from Yakutat Bay over a seven-year period shared the same haplotype, but here many were also closely related (Fig 7G). This may not be too surprising because this geographically isolated group numbers less than 20 individuals and is relatively inbred^12^.

A third case may be the first indication that in certain situations, or perhaps locations, there are preferential associations among maternal relatives and multiple such matrilineal groups may at times associate. Within a mixed-age herd from Husky Lakes individual whales that possessed the same haplotype had higher mean relatedness to each other (Fig 5C) and tended to cluster together within the network of genetic relationships (Fig 7E). The herd was one of the few instances when dependent calves were sampled and, interestingly, they tended to play a central role in the genetic network (Fig 7E), reflecting possible inter-familial breeding.

*The nature of the larger adult-male groups, and of adult-male herds in beluga whales*

The small adult male groups in beluga whales were likened to dolphin alliances in the main text. The larger adult-male groups may indicate a hierarchy of alliance coalescence in beluga whales, as has been described for bottlenose dolphins in Shark Bay, Australia^6,7^, or perhaps looser ‘bachelor’ groupings that may emerge outside the breeding season, or when other age and sex classes are engaged in other activities (e.g., natal care and play).

Ecological rather than reproductive drivers may be more important in the formation of adult-male herds compare to the smaller adult-male social groups. These herds, some numbering several hundred, were spatially segregated from other group types, were observed migrating *en masse* in spring, and may remain together during the summer. Age and sex segregation in beluga whales have been observed by others (e.g., ^16,17,4,18,19,20^). Adult male belugas are larger than all other age and sex categories^16,20^. Michaud^18^ found that ecological factors and physical characteristics were the most likely primary drivers of spatial sexual segregation in sexually dimorphic odontocetes, including beluga whales, where predation risks, energetic requirements, and habitat needs likely differ between adult males and the smaller adult females and immatures. Loseto et al.^19^ linked evidence of spatial sex segregation in satellite tracked beluga whales in the Beaufort Sea to likely ecological differences. Large male groupings are also less likely to be reproductive in nature than smaller ones because the likely reproductive advantage to the individual within the larger grouping is lower^9^. For these same reasons, individuals within the larger herds may be less motivated to be cooperative.

**Literature cited**

1. Queller, D. C. & Goodnight, K. F. Estimating relatedness using molecular markers. *Evolution* **43**, 258–275 (1989).
2. Blouin, M., Parsons, M., Lacaille, V. & Lotz, S. Use of microsatellite loci to classify individuals by relatedness. *Mol. Ecol.* **5**, 393–401 (1996).
3. Kraemer, P. & Gerlach G.Demrelate: calculating interindividual relatedness for kinship analysis based on codominant diploid genetic markers in R. *Mol. Ecol. Resour.* **17**, 1371-1377 (2017).
4. Smith, T. G., Hammill, M. O. & Martin, A. R. Herd composition and behaviour of white whales (*Delphinapterus leucas*) in two Canadian arctic estuaries. *Meddelelser om Grønland, Bioscience* **39**, 175-184 (1994).
5. Krasnova, V. V., Chernetsky, A. D., Zheludkova, A. I. and Bel’kovich, V. M. Parental behavior of the beluga whale (*Delphinapterus leucas*) in natural environment. *Biol. Bull*. **41**, 349-356 (2014).
6. Connor, R.C., Wells, R., Mann, J. and Read, A. The bottlenose dolphin: social relationships in a fission-fusion society. In *Cetecean Societies: Field Studies of Dolphins and Whales* (eds Mann, J., Connor, R. C., Tyack, P. L. & Whitehead, H.) 91-126 (The Univerisity of Chicago Press Ltd., 2000).
7. Connor, R. C., Cioffi, W. R., Randić, S., Allen, S. J., Watson-Capps, J. & Krützen, M. Male alliance behaviour and mating access varies with habitat in a dolphin social network. *Sci. Rep*. **7**, 46354; DOI: 10.1038/srep46354 (2017).
8. Packer, C., Gilbert, D. A., Pusey, A. E. & O’Brien, S. J. A molecular genetic analysis of kinship and cooperation in African lions. *Nature* **351**, 562-565 (1991).
9. Grinnell, J., Packer, C. & Pusey, A.E. Cooperation in male lions: kinship, reciprocity or mutualism? *Anim. Behav*. **49**, 95-105 (1995).
10. Maynard Smith, J. Game theory and the evolution of cooperation. In: *Evolution from Molecules to Men* (ed. Bendall, D. S.) 445-456 (Cambridge University Press, 1983).
11. Trivers, R. L. The evolution of reciprocal altruism. *The Quarterly Review of Biology* **46**, 35-57 (1971).
12. O’Corry-Crowe, G., Lucey, W., Archer, F. I. & Mahoney, B. The genetic ecology and population origins of the beluga whales of Yakutat Bay. *Mar. Fish. Rev.* **71**, 47-48 (2015).
13. deMarch, B. G. E. & Postma, L. D. Molecular genetic stock discrimination of belugas (*Delphinapterus leucas*) hunted in eastern Hudson Bay, northern Quebec, Hudson Strait, and Sanikiluaq (Belcher Islands), Canada, and comparisons to adjacent populations. *Arctic* **56**, 111-124 (2003).
14. Colbeck, G. J., Duchesne, P., Postma, L. D., Lesage, V., Hammill, M. O. & Turgeon, J. Groups of related belugas (*Delphinapterus leucas*) travel together during their seasonal migrations in and around Hudson Bay. *Proc. R. Soc. B*. **280**, 2012-2552 <https://doi.org/10.1098/rspb.2012.2552> (2013).
15. O’Corry-Crowe, G., Suydam, R., Quakenbush, L., Potgieter, B., Harwood, L., Litovka, D., Ferrer, T., Citta, J., Burkanov, V., Frost, K. & Mahoney, B. Migratory culture, population structure and stock identity in North Pacific beluga whales (*Delphinapterus leucas*). *Plos ONE* **13(3)**, e0194201 <https://doi.org/10.1371/journal.pone.0194201> (2018).
16. Kleinenberg, S. E., Yablokov, A. V., Bel’kovich, B. M. & Tarasevich, M. N. *Beluga (Delphinapterus leucas) Investigation of the Species*. (Academy of Sciences of the USSR, 1964).
17. Caron, L. M. J. & Smith, T. G. Philopatry and site tenacity of belugas, *Delphinapterus leucas*, hunted by the Inuit at Nastapoka estuary, eastern Hudson Bay. In: *Advances in Research on the Beluga Whale, Delphinapterus leucas* (eds. Smith, T. G. St. Aubin, D. J. & Geraci, J. R.) 69-79. *Can. Fish. Aquatic Sci.* **224** (1990).
18. Michaud, R. Sociality and ecology of the odontocetes. In: *Sexual Segregation in Vertebrates: Ecology of the Two Sexes* (eds. Ruckstuhl, K. E. & Neuhaus, P.) (Cambridge University Press, 2005).
19. Loseto, L. L., Richard, P., Stern, G. A., Orr, J. & Ferguson, S. H. Segregation of Beaufort Sea beluga whales during the open-water season. *Can. J. Zool*. **84**, 1743-1751 (2006).
20. Suydam, R. S. *Age, growth, reproduction, and movements of beluga whales (Delphinapterus leucas) from the eastern Chukchi Sea. Ph.D. Thesis* (2009).
